# Size Distribution, Mechanical and Electrical Properties of CuO Nanowires Grown by Modified Thermal Oxidation Methods

**DOI:** 10.3390/nano10061051

**Published:** 2020-05-29

**Authors:** Raitis Sondors, Jelena Kosmaca, Gunta Kunakova, Liga Jasulaneca, Matiss Martins Ramma, Raimonds Meija, Edijs Kauranens, Mikk Antsov, Donats Erts

**Affiliations:** 1 Institute of Chemical Physics, University of Latvia, 1 Jelgavas str., LV-1004 Riga, Latvia; raitis.sondors@lu.lv (R.S.); jelena.kosmaca@lu.lv (J.K.); gunta.kunakova@lu.lv (G.K.); liga.jasulaneca@lu.lv (L.J.); matiss_martins.ramma@lu.lv (M.M.R.); raimonds.meija@lu.lv (R.M.); edijs.kauranens@lu.lv (E.K.); antsof@gmail.com (M.A.); 2 Department of Chemistry, University of Latvia, 1 Jelgavas str., LV-1004 Riga, Latvia

**Keywords:** CuO, nanowire, synthesis, thermal oxidation, Young’s modulus, electrical resistivity, NEMS

## Abstract

Size distribution, Young’s moduli and electrical resistivity are investigated for CuO nanowires synthesized by different thermal oxidation methods. Oxidation in dry and wet air were applied for synthesis both with and without an external electrical field. An increased yield of high aspect ratio nanowires with diameters below 100 nm is achieved by combining applied electric field and growth conditions with additional water vapour at the first stage of synthesis. Young’s moduli determined from resonance and bending experiments show similar diameter dependencies and increase above 200 GPa for nanowires with diameters narrower than 50 nm. The nanowires synthesized by simple thermal oxidation possess electrical resistivities about one order of magnitude lower than the nanowires synthesized by electric field assisted approach in wet air. The high aspect ratio, mechanical strength and robust electrical properties suggest CuO nanowires as promising candidates for NEMS actuators.

## 1. Introduction

Metallic, semiconducting and insulating Cu based nanomaterials have been demonstrated and are well recognized materials in the fields of electronic devices and catalysis. Metallic nanowires of Cu have mostly been used for applications in flexible and transparent electrode materials. Cu nanowires have been synthesized by several approaches, for example, the hydrothermal reduction process [1] and template-based electrodeposition [2], as well as the thermal reduction of copper(II) oxide CuO nanowires [3].

Semiconducting Cu-based nanowires, such as p-type CuO in particular, are commonly recognized as a catalyst material [4]. Nanowires of CuO have attracted broad interest in many applications including in sensors [5,6,7] and nanoelectronics (transistors [8,9], electric field emitters [10], memristors [11] and other), mainly due to their enhanced aspect ratio [12], achieved by using relatively simple and low cost synthesis. CuO nanowires also exhibit high stiffnesses [13], which make them promising candidates for high-frequency and speed nanoelectromechanical systems (NEMS) [14,15]. Nanoresonators [16] and switches [15,17] employ nanowires for both mechanical actuation and electrical signal transduction. To achieve improved performance of these devices, more extensive studies of mechanical and electrical properties of the nanowires and the development of simple growth protocols to controllably achieve nanowires with required parameters are needed.

The growth of CuO nanowires can be realized through various routes; for example, hydrothermal [18], electrochemical [19], solid-state conversion [20] or thermal oxidation [12]. Thermal oxidation is considered as one of the simplest and cheapest methods for scalable synthesis of crystalline CuO nanowires [12]. In this process, the surface of Cu substrates becomes oxidized and forms a Cu_2_O layer, which acts as a precursor for the formation of a CuO layer with nanowires. Previous studies revealed that synthesis parameters (temperature, time, air flow, etc.) can significantly affect both the yield and size distribution of the nanowires [21,22]. The synthesis typically utilizes a temperature range from 400 °C to 700 °C [12,23]. Nanowire synthesis in the presence of water vapour resulted in longer and thinner nanowires due to an increased oxidation rate of copper [24]. A similar effect was observed for nanowire synthesis in external electric field [25], where CuO nanowires grew longer [26] and had more uniform diameters [25]. However, a combination of both humidity and electric field during CuO nanowire synthesis by thermal oxidation has yet to be studied. Moreover, the crystalline structure of CuO nanowires, which is impacted by the synthesis method, may also influence their mechanical properties [13]. 

The mechanical and electrical characterization of nanowires is challenging, since it requires precise manipulation at the nanoscale. The Young’s moduli must be carefully evaluated by measuring individual nanowires, because they can exhibit size-dependence arising from defects [13] and surface stress [27,28]. Previously reported Young’s moduli for CuO nanowires synthesized by thermal oxidation were in the range of 70–300 GPa [13], and showed a significant increase for thin nanowires compared to bulk material [13]. The electrical transport mechanisms of similar nanowires contacted by metallic electrodes can be a combination of ohmic with resistivities of 10–40 Ωm [9,23], and space-charge-limited current (SCLC) [29,30], similar to other semiconductor nanowires [31,32]. Electrical properties of CuO nanowires were also observed to depend significantly on the defect concentrations [30].

In this work, we have investigated the electric field-assisted thermal oxidation of Cu foil to achieve a high yield growth of high aspect ratio CuO nanowires in conditions of wet air, and compared the properties of these nanowires with those synthesized by a simple thermal oxidation in dry air. Statistical analysis of the experimentally measured geometrical parameters of the nanowires grown by different methods and synthesis conditions (dry and wet air) was used to compose detailed distributions of the nanowire geometrical parameters. The Young’s moduli were determined from mechanical resonance by in-situ electron microscopy [33,34] and bending tests by atomic force microscopy [33,35], which have proven suitability for assessing the properties of individual nanowires. Current-voltage characteristics were recorded for individual CuO nanowires of different lengths between the electrodes and the transfer length method was used to determine contact resistances and calculate the resistivities of the CuO nanowires. The determined characteristics of the nanowires were compared to properties of other nanomaterials, which have found application in NEMS.

## 2. Materials and Methods 

**Figure 1:**
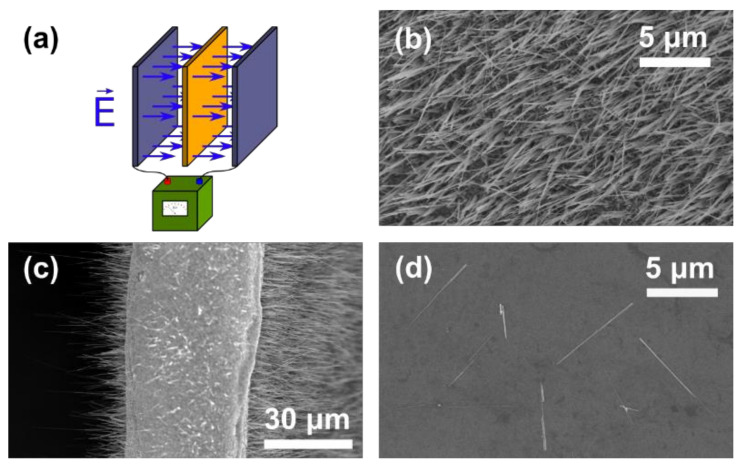
(**a**) Schematic representation of Cu foil between electrodes; SEM images: (**b**) CuO nanowires synthesized on a Cu foil (top view); (**c**) CuO nanowires synthesized on a Cu foil (side view); (**d**) transferred CuO nanowires on Si/SiO_2_ substrate.

Synthesis of the CuO nanowires on a Cu foil substrate (99.9% purity, 25 μm thickness) (Goodfellow GmbH, Hamburg, Germany) was performed in a GSL-1100X tube furnace (length 60 cm, diameter 46 mm) (MTI Corporation, Richmond, CA, USA). To prepare the substrates, the foil was cut in 16 × 25 mm pieces and rinsed in distilled water. The substrates were placed in the furnace tube and heated from room temperature up to 500 °C for 30 min at a constant heating rate of 16 °C/min. The temperature was maintained constant at 500 °C for 210 min, after which the furnace was turned off and left to cool down to room temperature. To increase air humidity during the initial stage of synthesis, a small vessel containing 7 μL of distilled water was placed inside the furnace tube near the substrate. This was done when the temperature had reached the critical temperature of water (374 °C) to avoid the formation of water droplets on the substrate. For generation of electric field, the Cu foil was placed between two steel electrodes 1 cm apart, and a voltage of 200 V was applied by a Keithley 6487 Picoammeter/Voltage source (Tektronix (China) Co. Ltd, Shanghai, China) (Figure 1a). 

After synthesis, the oxidized foil was examined in a Hitachi S4800 scanning electron microscope (SEM, Hitachi Ltd., Chiyoda, Tokyo, Japan). The obtained images confirmed that nanowires had grown on both sides of the foil substrate (Figure 1b). The oxidized surface layer of foil containing synthesized nanowires was then mechanically removed and dispersed in isopropanol via ultrasonication for 3 s. The layer containing nanowires was removed from both the cathode and anode sides separately for the electric field assisted synthesis (Figure 1c).

The dispersed nanowires were then transferred to a Si/SiO_2_ chip by placing a drop of the suspension on the substrate. After the isopropanol had evaporated the nanowires were examined in a SEM. The acquired images (Figure 1d) were analyzed with ImageJ software to determine nanowire lengths and diameters. SEM images with typical magnifications of ×1k and ×90k were used for the measurements of lengths and diameters, respectively. The measured length and diameter data were organized into separate datasets for each combination of synthesis parameters and used for extraction of the mean values and histograms.

**Figure 2:**
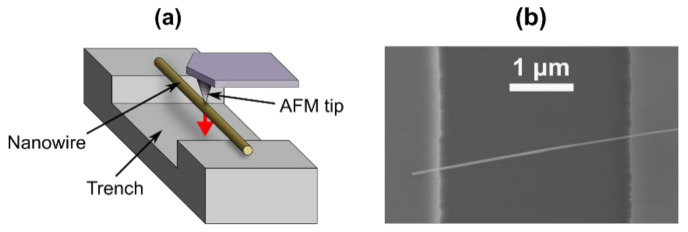
(**a**) Schematic of the AFM 3-point bending test; (**b**) SEM image of a typical CuO nanowire on a trench (top view).

Mechanical characterization of the nanowires was performed by 3-point bending tests on double-clamped nanowires by a Bruker Dimensions Edge atomic force microscope (AFM) (Bruker Corporation, Billerica, MA, USA) and mechanical resonance tests on single-clamped nanowires by in situ SEM [33,35]. For the bending tests, nanowires were transferred onto chemically etched trenches with heights of 0.5 µm and widths of 6 µm prepatterned on SiO_2_ substrate surface by gently pressing the Cu foil against it. This resulted in randomly placed nanowires, and freely suspended nanowires with both ends supported were chosen for 3-point bending tests. Figure 2 shows a schematic of the AFM 3-point bending test and an SEM picture of a single nanowire on a trench. A number of force-displacement curves were measured, and Young’s moduli were calculated by using finite element method (FEM) [35].

**Figure 3:**
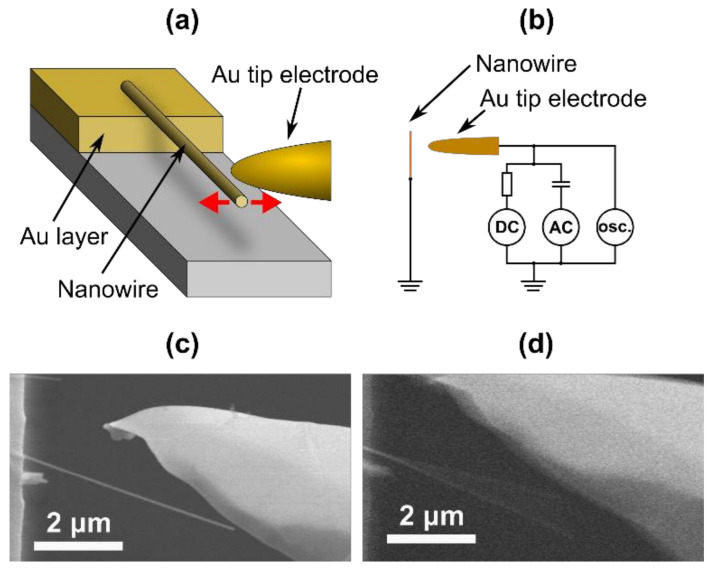
(**a**) Schematic of the in-situ SEM resonance test; (**b**) electric circuit during the resonance test; (**c**) CuO nanowire before resonance; (**d**) CuO nanowire during resonance.

For the resonance tests, nanowires in the isopropanol suspension were dielectrophoretically aligned [36,37] on the edges of pre-patterned Cr/Au electrodes with heights of 0.5 µm above the SiO_2_ substrate surface. Aligned nanowires with one end supported on the electrode and the other end suspended were chosen for the resonance measurements. Nanowire vibrations were excited by AC/DC voltage applied between the nanowire and a metallic tip counter electrode (Figure 3a,b). The AC signal was generated by the AC sweep function generator Agilent N9310A (Agilent Technologies Inc., Santa Clara, CA, USA), the DC voltage was supplied by a Keithley 6487 voltage source, the signal was monitored by a TDS 1012 oscilloscope (Tektronix Inc., Beaverton, OR, USA). The resonance was detected visually from SEM images (Figure 3c,d). The Young’s moduli were determined from nanowire resonance frequencies and geometry analytically using Euler–Bernoulli theory.

To enable electrical transport measurements, CuO nanowires were mechanically transferred by gently pressing a peeled-off copper oxide layer on Si/SiO_2_ substrates. Electron beam lithography was used to fabricate contacts to the individual nanowires. Sequential layers of Pd (3 nm) and Au (80 nm) were evaporated as the electrode material. Current–Voltage characteristics (IVC) were recorded at ambient conditions using a Keithley 4200SCS parameter analyser (Keithley Instruments, Inc., Cleveland, OH, USA).

## 3. Results and Discussion

### 3.1. Size Distribution of CuO Nanowires

The geometrical parameters of CuO nanowires (diameter and length) were determined using SEM images captured for nanowires transferred to Si/SiO_2_ substrates. In total, more than eight thousand measurements of length and diameter were recorded and summarized in datasets. Statistical analysis was applied to evaluate the distributions of the nanowire dimensions in histograms and extract the mean values of diameters and lengths for each type of synthesis.

**Figure 4:**
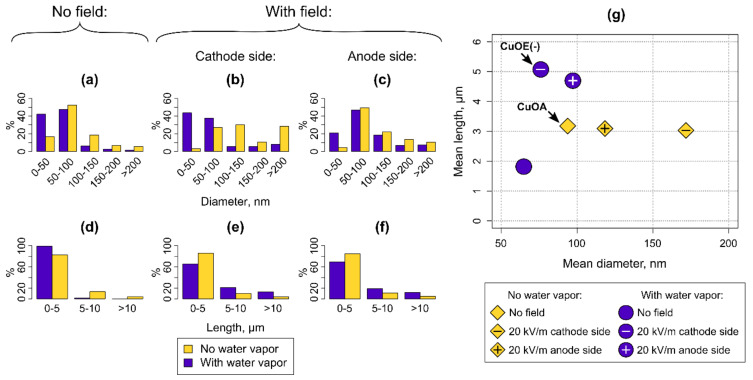
(**a**–**c**) Diameters and (**d**–**f**) lengths of CuO nanowires: (**a**) and (**d**) with no electric field; (**b**) and (**e**): with an applied 20 kV/m electric field on the cathode side of the substrate; (**c**) and (**f**) with an applied 20 kV/m electric field on the anode side of the substrate; (**g**) mean diameters and lengths of CuO nanowires synthesized by different methods. The nanowires selected for the mechanical and electrical characterization are denoted as CuOA—nanowires synthesized by thermal oxidation (dry air), CuOE(-)—nanowires synthesized in electric field (cathode side, wet air).

Figure 4a–f shows histograms of the diameters and lengths of nanowires synthesized by thermal oxidation with and without electric field and in wet or dry air. Diameters of the nanowires were found to be in a range from 20 to 320 nm for all the growth approaches and conditions used, with some of the thinnest nanowires reaching 5 nm in diameter (Figure 4a–c). However, these diameter distributions show a different percentage of nanowires with diameters below 100 nm for different growth approaches.

(1) Thermal oxidation without electric field. The synthesis in normal dry air conditions resulted in the relatively narrow, symmetrical distribution of nanowire diameters mainly being below 100 nm. Approximately 69% of the nanowires synthesized by this method were thin (<100 nm) (Figure 4a). Conditions of added water vapour resulted in notably increased ratio of the small diameter nanowires (50 nm and below) and increased the proportion of the thin nanowires up to 89% (Figure 4a). This phenomenon could be explained considering the fact that the water vapour initially increases the oxidation rate of copper [24], which is particularly important at the beginning of the growth. Under these conditions, grain size of CuO is expected to increase and eventually slow down the further oxidation of copper [38,39]. When the content of water vapour becomes reduced, further growth of the CuO nanowires takes place without the presence of the water vapour resulting in nanowires of narrower diameters. 

(2) Thermal oxidation with electric field. The synthesis employing electric field yielded in broader distributions of nanowire diameters, compared to the results from conventional synthesis (Figure 4g). The nanowires can be obtained on both the cathode and anode sides of the substrate, and the nanowires from these sides were found to have different size distributions (Figure 4b,c). Obtained distributions of diameters of the nanowires from the substrate surface at the anode side were rather similar in both conditions of the synthesis (in dry and wet air, Figure 4c,g) and comparable to the ones synthesized without electric field (Figure 4a,g). Approximately half of the nanowires grown on the anode side of the substrate were with diameters of 50–100 nm (Figure 4c). 

However, the distribution of the nanowire diameters obtained from the cathode side of the substrate is entirely different. Here the growth conditions more prominently affect the nanowire diameters, and wider distribution of dominating 100–150 nm, and >200 nm diameter nanowires were achieved for synthesis without water vapour (Figure 4b,g). As a result, the number of thin nanowires dropped from 69% down to 34%. Interestingly, nearly the opposite trend (narrower distribution with dominating thin <50 nm and 50–100 nm diameters of the nanowires) can be achieved if the water vapour is used (Figure 4b,g). The percentage of thin nanowires of 81% on the cathode side is close to that in wet air thermal oxidation without electric field (89%), and suggests that humidity compensates the effect of electric field on nanowire diameter.

The lengths of the nanowires transferred from the synthesis substrate to surface of SiO_2_ varied from 0.2 to 39 µm. The nanowires became shorter due to breakage during the ultrasonication process. Thinner nanowires are expected to break more than the thicker ones. This can explain why the nanowires with the smallest mean diameter have the shortest mean length (Figure 4g). Most of the transferred nanowires synthesized within all the approaches and conditions used were of lengths below 5 µm (Figure 4d–g). The number of nanowires with lengths >5 µm increased for the electric field assisted synthesis (Figure 4e–g), particularly within conditions without the water vapour. 

The different diameter distribution on the cathode and anode sides could be due to the effect of the electric field on Cu+ ion diffusion direction and velocity. The growth mechanism is similar on both sides of the substrate placed between the cathode and anode electrodes [25,26,40]; therefore, the composition of the nanowires grown on both sides is expected to be similar. The electric field influences lengths and thicknesses of the nanowires, because it facilitates Cu and O ion diffusion along the growth direction of the nanowires parallel to the external electric field. The effect of electric field is more pronounced on the cathode side, since the direction of the external electric field matches the local electric field set up by the O ions at the solid/gas interface. This promotes the Cu ion diffusion and results in increased mass of the CuO nanowires, manifested through increased diameters (synthesis in dry air) or increased lengths (synthesis in wet air). Our results support previously observed changes in diameters and increased lengths and yields of the nanowires grown with electric field compared to the nanowires grown without electric field [25,26].

### 3.2. Mechanical Characterization

For the fabrication of NEM devices, longer nanowires with diameters below 100 nm are preferable, because they provide lower switch-on voltages and lower adhesion in the contact [41]. The best characteristics for application in NEMS are demonstrated for nanowires synthesized by simple oxidation of Cu in dry air (mean diameter appr. 90 nm and mean length 3 µm) and synthesized with the assistance of electric field on the cathode in a wet air atmosphere (mean diameter appr. 70 nm and mean length 5 μm) (Figure 4g). Nanowires from both batches were chosen for further mechanical and electrical characterization and in further text are denoted as CuOA (thermal oxidation in dry air) and CuOE(-) (electric field, oxidation on the cathode side, wet air). 

**Figure 5:**
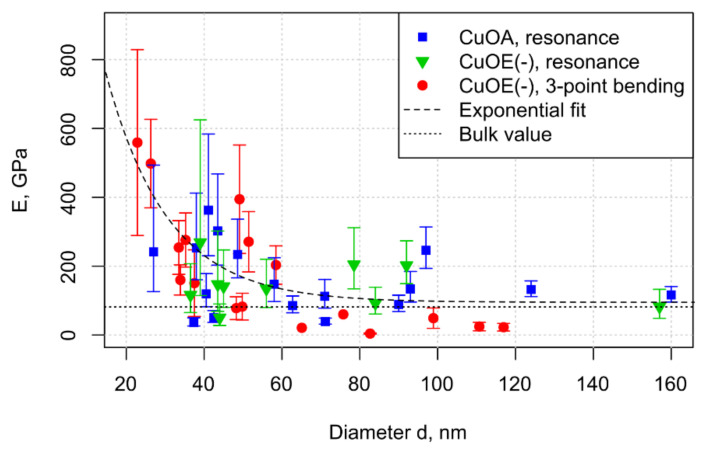
Experimental Young’s moduli from resonance and bending tests obtained for 44 CuO nanowires vs. their mean diameters; CuOA—nanowires synthesized by thermal oxidation (dry air), CuOE(-)—nanowires synthesized in electric field (cathode side, wet air). The dashed line is a guide to the eye; the dotted line is the bulk Young’s modulus determined in [13].

The Young’s moduli were found to be similar for the CuOA and CuOE(-) nanowires. Moreover, the Young’s moduli determined from the AFM 3-point bending tests are in agreement with in-situ SEM resonance tests (Figure 5). The observed trend of the Young’s moduli for the diameter range 20–160 nm can be described by an exponential trendline (Figure 5, dashed line). When increasing nanowire diameter, the exponential trendline approaches 95 GPa, which is close to the value of 82 GPa for bulk CuO [13]. The size-dependence becomes pronounced for nanowires with diameters below 50 nm. Such size-dependent behavior is in agreement with the previously reported experimental data on CuO nanowires synthesized by thermal oxidation in dry air [13]. The size-dependence of the nanowire Young’s modulus can be attributed to the effects of tensile surface stresses [42], which depends on the loading mode (static bending/dynamic resonance) and boundary conditions of the nanowire [27,28]. This can explain a more pronounced size dependence in Young’s moduli from bending than resonance. The highest Young’s moduli determined for 20–30 nm diameter CuO nanowires can reach even up to ~500 GPa, which is comparable to the Young’s moduli for NEMS active element materials, such as TiN and SiC [15].

### 3.3. Electrical Characterization

**Figure 6:**
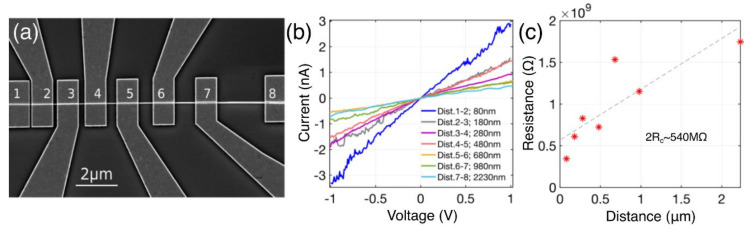
(**a**) SEM image of CuO nanowire with 8 electrodes of distances from 80–2380 nm; (**b**) current—voltage characteristics measured at room conditions and in 2-probe configuration; (**c**) resistance as a function of the nanowire length (different distance between the electrodes). Linear fit (dashed line) intercepts with the y axis at around 540 MΩ representing the contact resistance of the two electrodes 2Rc. SEM image and all the data shown here were recorded for CuO nanowire grown in dry air, without electrical field, nanowire CuOA–D1.

**Figure 7:**
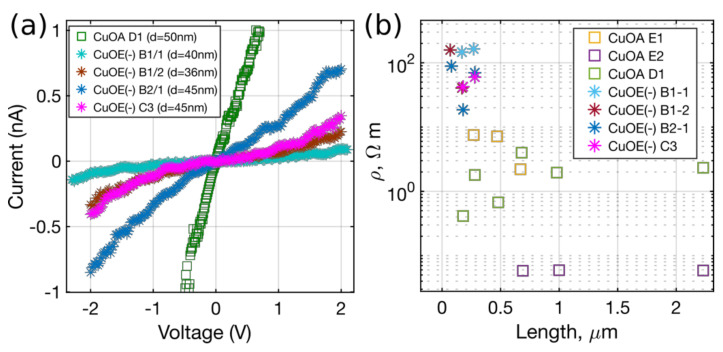
(**a**) IVCs of the CuO nanowires grown via two different approaches; (**b**) calculated resistivities *ρ* of CuO nanowires shown as a function of the measured nanowire length (distance).

A SEM image of one of the CuO nanowires with electrodes fabricated by e-beam lithography for four-point measurements is shown in Figure 6a. In total, seven nanowires—three of them grown by oxidation in dry air CuOA and the others via electric field assisted approach in the presence of added water—were incorporated into similar multiple-contact devices with the same distances between the electrodes. IVCs for the nanowire CuOA–D1 with diameter of 50 nm are plotted in Figure 6b. These IVCs were recorded in a two–electrode configuration, which also includes the contribution from contact resistance, and in the measurements a voltage range of ±2 V (Figure 7a) was used. The voltage range of ±1 V, where the IVCs were linear for all the measured nanowire lengths, was used to extract the resistance.

The resistance of the contacts to a single nanowire can be evaluated by the transfer length method where the total measured resistances are expected to scale with the nanowire lengths. Resistance as a function of the distance is shown in Figure 6c. The linear fit intercepts with the resistance axis at 540 MΩ, which represents resistance of the contacts 2R_c_. This extracted value of R_c_ is rather high and more effort of CuO nanowire contact interface engineering is needed to achieve lower contact resistances. The extracted values of R_c_ were of the MΩ range for all the CuOA nanowires-540 MΩ (CuOA-D1); 330 MΩ (CuOA-E1) and 6.63 MΩ (CuOA-E2). The CuOE(-) nanowires showed far larger resistances especially for the long nanowire lengths (contact pairs 5–6, 6–7, 7–8, see SEM image, Figure 6a) making it impossible to obtain reasonable resistance–distance dependences. In the further calculations of resistivities for CuOE(-) nanowires, the largest determined contact resistance of 540 MΩ was accounted for.

IVCs recorded for nanowires of the same electrode separation (~180 nm, electrodes 2–3 in Figure 6a) and of diameters ranging from 36 to 50 nm are depicted in Figure 7a. In order to compare the two kinds of CuO nanowires, resistivities for all the measured nanowires were calculated as *ρ* = (R − 2R_c_)A/*l*, where A and *l* are the cross-section area and the length of the nanowire, respectively.

Calculated resistivities for all the nanowires are plotted as a function of the distance (see Figure 7b). In the case of a uniform nanowire/contact resistance the resistivity should be the same for all the nanowire lengths in terms of a single nanowire. This case was entirely true for the CuOA–E1 nanowire of diameter 50 nm (violet squares, Figure 7b), but for the others the values of resistivity vary slightly for different nanowire lengths indicating nonuniform contact (nanowire) resistances. As one can see, the calculated resistivities of CuOA nanowires (0.05–7 Ωm) are lower compared to the ones extracted for CuOE(-) nanowires (20–160 Ωm). This could be explained assuming increased defect concentration [30] for the nanowires synthesized with the electric field assisted approach and in presence of water vapour. Despite the differences in resistivity for CuOA and CuOE(-) nanowires, in general, the resistivities of both types of nanowires are comparable to previously reported values of 10–40 Ωm for CuO nanowires synthesized by simple thermal oxidation method and measured in field effect transistor geometries [9,23]. These values match with characteristics of some materials applied in previously demonstrated NEM switch designs; for example, semiconductor Si and SiC [15]. The relatively high resistivities may be related to low mobility of charge carriers in CuO nanowires at room temperature [29]. The mobility can be increased by increasing the temperature. Due to a high melting point, CuO nanowires are able to withstand harsh environments, similarly to previously demonstrated high-temperature SiC switches [43]. Alternatively, such CuO nanowires are attractive for NEM switches and other devices with high operating voltages, where space-charge-limited currents are expected to dominate charge carrier transport in the nanowires [29,30,32].

## 4. Conclusions

In summary, CuO nanowires synthesized by a simple thermal oxidation and electric field assisted approach were investigated for different synthesis conditions (in dry and wet air). High yields of large aspect ratio nanowires with diameters below 100 nm were achieved by increasing air humidity during the initial step of the electric field assisted synthesis. Nanowires synthesized by simple thermal oxidation in dry air and by electric field assisted approach in wet air exhibit similar mechanical properties. Nanowires with diameters less than 50 nm show higher Young’s moduli of up to 200–500 GPa. The electrical resistivities are comparable with previously reported values for CuO nanowires, and show an increase for the nanowires synthesized by thermal oxidation with electric field in wet air. Relatively high electrical resistivities in combination with high mechanical stiffness and a high melting point can be favorable in NEM-switching devices operating in extreme conditions (harsh environments and high applied voltages).
